# Better assessment of physical function: item improvement is neglected but essential

**DOI:** 10.1186/ar2890

**Published:** 2009-12-16

**Authors:** Bonnie Bruce, James F Fries, Debbie Ambrosini, Bharathi Lingala, Barbara Gandek, Matthias Rose, John E Ware

**Affiliations:** 1 Department of Medicine, Stanford University School of Medicine, 1000 Welch Road, Suite 203, Stanford, CA 94304, USA; 2 QualityMetric Inc, 640 George Washington Highway, Suite 201, Lincoln, RI 02865, USA; 3 Department of Psychosomatic Medicine, University Medical Center Hamburg-Eppendorf, Martinistrasse 52, 20246 Hamburg, Germany

## Abstract

**Introduction:**

Physical function is a key component of patient-reported outcome (PRO) assessment in rheumatology. Modern psychometric methods, such as Item Response Theory (IRT) and Computerized Adaptive Testing, can materially improve measurement precision at the item level. We present the qualitative and quantitative item-evaluation process for developing the Patient Reported Outcomes Measurement Information System (PROMIS) Physical Function item bank.

**Methods:**

The process was stepwise: we searched extensively to identify extant Physical Function items and then classified and selectively reduced the item pool. We evaluated retained items for content, clarity, relevance and comprehension, reading level, and translation ease by experts and patient surveys, focus groups, and cognitive interviews. We then assessed items by using classic test theory and IRT, used confirmatory factor analyses to estimate item parameters, and graded response modeling for parameter estimation. We retained the 20 Legacy (original) Health Assessment Questionnaire Disability Index (HAQ-DI) and the 10 SF-36's PF-10 items for comparison. Subjects were from rheumatoid arthritis, osteoarthritis, and healthy aging cohorts (n = 1,100) and a national Internet sample of 21,133 subjects.

**Results:**

We identified 1,860 items. After qualitative and quantitative evaluation, 124 newly developed PROMIS items composed the PROMIS item bank, which included revised Legacy items with good fit that met IRT model assumptions. Results showed that the clearest and best-understood items were simple, in the present tense, and straightforward. Basic tasks (like dressing) were more relevant and important versus complex ones (like dancing). Revised HAQ-DI and PF-10 items with five response options had higher item-information content than did comparable original Legacy items with fewer response options. IRT analyses showed that the Physical Function domain satisfied general criteria for unidimensionality with one-, two-, three-, and four-factor models having comparable model fits. Correlations between factors in the test data sets were > 0.90.

**Conclusions:**

Item improvement must underlie attempts to improve outcome assessment. The clear, personally important and relevant, ability-framed items in the PROMIS Physical Function item bank perform well in PRO assessment. They will benefit from further study and application in a wider variety of rheumatic diseases in diverse clinical groups, including those at the extremes of physical functioning, and in different administration modes.

## Introduction

Physical function is a key component of patient-reported outcome (PRO) assessment in the rheumatic diseases. Valid and meaningful PRO assessment requires instruments that are clear, relevant, and psychometrically robust. Traditional PRO instruments, like the 20-item Health Assessment Questionnaire Disability Index (HAQ-DI) [1,2] and the SF-36 10-item Physical Function scale (PF-10) [3], have been widely used to assess functional status in clinical trials and observational studies. Both instruments have been extensively validated by using traditional methods. However, these Legacy instruments have not benefited from application of modern psychometric methods, such as Item Response Theory (IRT) [4,5] and Computerized Adaptive Testing (CAT) [6].

IRT quantitatively establishes item-measurement properties and identifies items with the highest information content. It results in measures that are more reliable and practical over a wide range of score levels [4,7]. In IRT, levels of the latent trait (for example, Physical Function) depend on an individual's item-level responses rather than on instrument scores [8,9]. An item's properties ultimately characterize an individual, and become an estimate of his or her unique functional status. IRT is used for developing short-form instruments and enabling CAT-based assessment.

CAT enables the tailoring of items to an individual (for example, their level of functioning) by sequentially selecting items that provide the maximal amount of information based on their previous responses [6]. It can reduce respondent burden, yield precise individual scores, and provide immediate feedback.

The National Institutes of Health Roadmap Initiative, the Patient Reported Outcomes Measurement Information System (PROMIS) [10], is directed at improving PROs and promoting their use by the public and private sectors. A major objective of PROMIS is to establish a national resource for accurate and efficient PRO measurement. PROMIS goals include creating a repository of validated items and short forms and a CAT system. Such tools will yield improved instruments with better and higher individualized information, resulting in increased study power with reduced sample sizes [9].

PROMIS Network investigators from six primary research sites developed a health-domain framework with the major dimensions of Physical, Mental, and Social health, by following that of the World Health Organization [11]. The initial set of PROMIS domains are Physical Function, Pain Impact and Behavior, Fatigue, Emotional Distress (Anxiety, Depression, Anger), and Social Health [12].

In addition, to enable a broader evaluation of health, PROMIS developed global items for each of the domains to assess general health rather than specific elements of health. For Physical Function, the global health item asks, "To what extent are you able to carry out your everyday physical activities, such as walking, climbing stairs, carrying groceries, or moving a chair," and is scored on a 5-point scale, ranging from "completely" to "not at all."

In PROMIS, the Physical Function latent trait is defined as the "ability to carry out various activities that require physical capability, ranging from self-care (basic activities of daily living (ADL)) to more-vigorous activities that require increasing degrees of mobility, strength, or endurance" [12]. Items are framed in the present tense, with a capability stem and a corresponding capability response set (for example, "Are you able to ..." from "no difficulty" to "unable to do"). Other physical-function items are constructed in terms of limitations due to health problems (for example, "Does your health now limit you..." from "not at all" to "cannot do"). A condition-specific attribution (such as rheumatoid arthritis) is not required to allow consistent estimation of physical function across different diseases and treatments and to avoid inaccuracies introduced by an attribution requirement.

The Physical Function domain is theoretically composed of four subdomains that are conceptually related but distinct. PROMIS proposes: mobility (lower extremity), dexterity (upper extremity), axial or central (neck and back function), and complex activities that involve more than one subdomain (instrumental activities of daily living). In practice, the subdomain assignment may sometimes be arbitrary, as many tasks involve more than one part of the body.

The purpose of this article is to present the qualitative and quantitative item-level evaluation process that was used to develop the PROMIS Physical Function item bank and the results of this evaluation.

## Materials and methods

**Figure 1:**
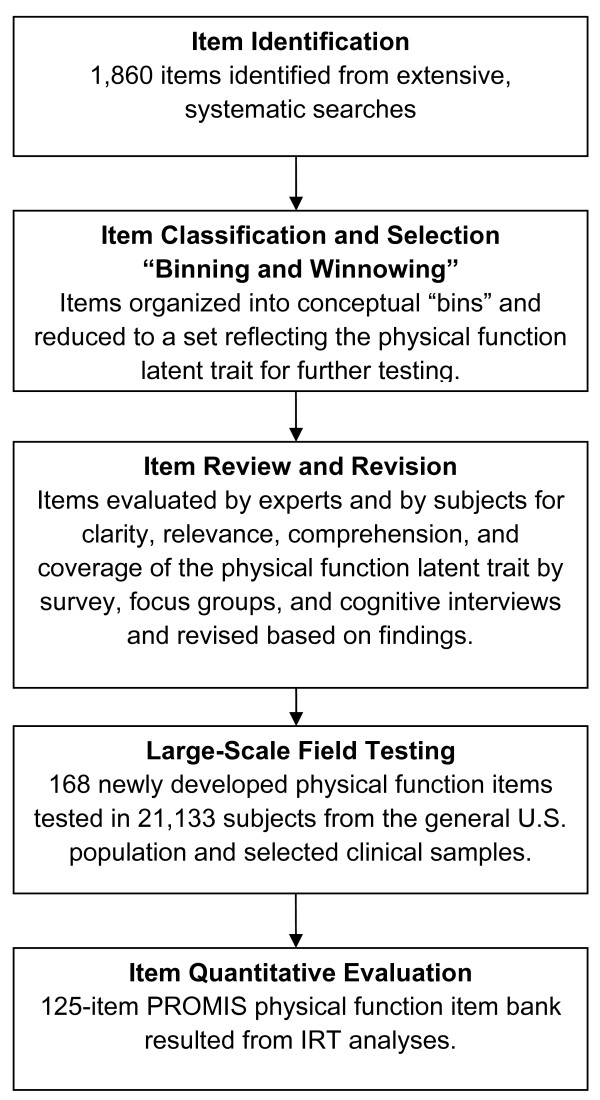
**Overview of the stepwise qualitative and quantitative activities used to develop the PROMIS Physical Function Item Bank**.

We followed systematic PROMIS Network-wide item-evaluation protocols [13]. All items underwent qualitative evaluation that identified strengths and problems based on assessments by expert and subject input, which was followed by quantitative analyses. Figure 1 presents an overview of the stepwise qualitative and quantitative activities used in development of the PROMIS Physical Function item bank.

The stepwise qualitative evaluation process consisted of: (a) identifying existing items; (b) classifying and selecting items (binning and winnowing); (c) initial item revision, including rewriting; (d) focus groups, aimed at identifying gaps in coverage of physical-function items and domain definition, and cognitive interviews, aimed at identifying potentially problematic items and helping to clarify physical-function items that were poorly understood and answered; and (e) final item revision. Items also were examined for reading level and translation ease.

Surviving items were then field-tested and underwent quantitative IRT analyses for item calibration and to create linking metrics to the Legacy HAQ-DI and PF-10 items. This study was approved by Stanford University's Human Subjects Research Protection Program (Protocol ID 10788), and each subject gave written informed consent.

### Subjects

Varying groups of subjects participated in the qualitative and quantitative evaluation activities.

#### Qualitative Item Evaluation Subjects

Item identification, classification, selection, and initial item revision were completed by project investigators (BB, JFF, DA, BG). For further evaluation of item clarity, relevance, and comprehension, subjects were drawn from the Stanford PROMIS Primary Research Site study cohort. They consisted of 1,100 ongoing participants from our four national, longitudinal studies: healthy seniors from the University of Pennsylvania [14] and the Precursors of Arthritis [15] studies (n = 550 (50%)) and rheumatoid arthritis (RA) (n = 275 (25%)) and osteoarthritis (OA) (n = 275 (25%)) subjects from the Arthritis, Rheumatism, and Aging Medical Information System [ARAMIS] [16]. On average, these subjects were white (87%), female (53%), and had an average of 16 ± 2.4 education years. Seniors from the University of Pennsylvania study were in their eighties, Precursors of Arthritis subjects were 72 years old, and subjects from ARAMIS averaged 67 years. RA and OA subjects had a mean disease duration of 24.6 ± 11.3 and 23.7 ± 12.3 years.

We conducted three focus groups with 15 subjects. Eight subjects with self-reported arthritis were recruited from a local arthritis self-management program. The remaining seven were recruited through flyers in clinics and on research bulletin boards, postings on websites and listserves, and through letters of invitation from a general medical rehabilitation population. The majority of the focus group subjects were female (80%) and white (93%). They ranged in age from 31 to 80 years old, and all had at least a high school education. Focus group subjects received a $20 gift card for participation.

For the cognitive interviews, 18 subjects from the study cohort completed semistructured telephone interviews. The majority were female (67%), white (67%), had at least a high school education (67%), and ranged in age from 48 to 93 years. These subjects received no compensation for participation.

#### Quantitative Item Evaluation Subjects

Items were field tested by 21,133 subjects. Ninety-three percent of the sample (n = 19,601) were recruited through Polymetrix, a polling firm based in Palo Alto, California [17]. The Polymetrix sample was designed to reflect Year 2000 United States census demographics. Two thirds of these subjects (n = 13,250) were drawn from the general United States population. The remaining Polymetrix subjects were recruited from Polymetrix clinical samples of individuals with rheumatoid arthritis (n = 557), osteoarthritis (n = 918), heart disease (n = 1,156), cancer (n = 1,754), psychiatric illness (n = 1,193), chronic obstructive pulmonary disease (n = 1,214), spinal cord injury (n = 531), and other conditions (n = 560). Data from Polymetrix subjects were collected by using their website on a secure server.

The remaining subjects were from the Stanford PROMIS Research Site study cohort and the North Carolina PROMIS primary research (n = 1,532). Data from these subjects were collected by using the PROMIS Assessment System [12].

To estimate the item parameters for Legacy HAQ-DI and PF-10 items, the Polymetrix sample was supplemented with subjects from the Stanford study cohort. These subjects received a small token, such as an easy-grip pen or $10, for participation.

Slightly more than half (52%) of the entire field-testing sample were women. Their median age was approximately 50 years. Sixty percent were at least 65 years old. The majority (82%) were white, and 97% had a high school education or higher. Field-tested subjects were included in the quantitative analyses if they had responded to 50% or more of the items, did not have repetitive strings of 10 or more identical responses, and their average online response time was greater than 1 second per item.

### Qualitative evaluation activities

#### Item Identification

We used an extensive and systematic strategy to identify extant English language self-report instruments. We used a very broad definition of physical function to enhance capture of instruments that at face value contained items that potentially measured some aspect of physical function. We searched for original articles and literature reviews over the past 30 years in PubMed and perused article bibliographies. We conducted broad Internet searches and examined various databases (such as the Patient Reported Outcomes Quality of Life Instruments Database (PROQOLID)). Our search terms included physical ability, function, functional status, limitations, disability, quality of life, self-report, and self-assessment questionnaire. For proprietary or copyrighted instruments, we used common PROMIS Network protocols and obtained permission to use physical-function items for development of the PROMIS Physical Function item bank.

#### Item Classification and Selection

We used a consensus-building process to organize items into conceptually similar and meaningful "bins" (for example, walking, dressing, hygiene). We then selectively winnowed the binned items to reduce the number to a workable set that reflected the Physical Function latent trait. We eliminated items that met the a priori criteria of being: (a) unrelated or inconsistent with the PROMIS definition of Physical Function; (b) confusing or unclear; (c) redundant or duplicated; (d) too condition specific; or (e) ungeneralizable.

#### Item Review and Revision

Subjects from the study cohort initially evaluated items for clarity, relevance, comprehension, and coverage of the physical function latent trait by mailed questionnaires. We developed 11 different questionnaires, with a maximum of 30 items each, to limit respondent burden. One hundred randomly selected subjects, consisting of 25 subjects from each of the four study cohorts, completed each questionnaire. The questionnaires included a mix of Physical Function items, performance ("Did you...") and capability framed ("Are you able to...") items, Legacy HAQ-DI and PF-10 items, and a few additional items designed to be purposely unclear, as a check on validity. Subjects rated candidate items for clarity ("Totally Clear", "Somewhat Clear", "Not Very Clear", or "Not Clear At All") and personal importance and relevance ("Very Important", "Somewhat Important", "Important", "A Little Important", or "Not Important at All"). They rated their preferences regarding item-recall period, response set, and best or worst response placement (for example, at one end of the scale or the other). To assess comprehension, we asked subjects to describe the meaning of selected items in their own words. The overall response rate was 73%. We then revised/rewrote items based on results and subject feedback.

For the focus groups, we used open-ended queries to encourage an interactive exchange. Each subject responded to six to nine items. We transcribed responses and created a summary that highlighted major themes.

For the cognitive interviews, we followed the PROMIS Network Cognitive Assessment Protocol. Each item was evaluated by three to six subjects who by protocol varied by gender, age (older than 65/65+) and race, and included at least one person with less than a high school education. For each item, we asked respondents: (a) if they had difficulty understanding the question (with further probes if they did); (b) what the question meant to them in their own words and how they would choose an answer (to test for comprehension and interpretation); (c) whether they would reword the question and, if so, how (to test for clarity and common language usage); (d) if the response choices were consistent with the question; and (e) if the question was easy or difficult to answer.

We tested 37 items, including 18 defined as problematic, in which more than 5% of subjects had rated the item as "Not Clear" or the item was "Totally Clear" for only 70% to 80% of the sample. We also asked each subject to evaluate: (a) the PROMIS global physical function item; (b) a sampling of performance items ("Did you..."); and (c) an item that had been judged "Very Clear" by all or virtually all raters to ensure that they understood the cognitive interview task correctly.

Based on subject feedback, we excluded items that were unclear to a large proportion of respondents and further revised/rewrote items, as indicated for quantitative analyses. The PROMIS statistical core also assessed items for readability at roughly the sixth-grade level and for translation ease. After this, items were discarded or, more frequently, revised to improve item quality.

After qualitative evaluation, the resulting Physical Function item bank consisted of 168 newly developed Physical Function items. The 20 Legacy HAQ-DI items and the 10 PF-10 items also were subjected to analyses.

#### Quantitative Analyses

We used psychometric techniques adopted by the PROMIS Network to examine properties of newly developed Physical Function items, the Legacy items, and the PROMIS Physical Function global health item [18-20]. Detailed descriptions of the quantitative IRT calibrations and simulated CAT testing of the PROMIS item banks have been published [7,18,19].

In short, we used factor analysis to examine underlying item structure and to assess IRT model assumptions. We conducted differential item function (DIF) analyses among chief demographic and clinical groups to examine items for differences, and we calibrated items to an IRT model for use in CAT. Greater detail about the PROMIS analytic plan is publicly available [21].

One of the main issues in developing the Physical Function item bank was exploration of its dimensionality. We began by analyzing all items by using the a priori assignment to the four Physical Function subdomains (upper extremity, lower extremity, axial, and complex activities). For all confirmatory factor analyses, we used polychoric correlations with the weighted least-squares mean and variance-adjusted estimator. As the four-factor solution showed very high correlations between all factors (*r *> 0.90), we investigated more parsimonious models, while retaining the four-factor model to improve content validity.

After confirming the assumptions of unidimensionality and local independence, we used a graded-response model (GRM) to estimate the item parameters. We excluded items with insufficient fit by using a SAS macro (SAS Institute, Cary, NC, USA) developed by Christensen and Bjorner [22]. We used a logistic regression approach for age, gender, and education to investigate DIF. An expanded discussion of empiric descriptions for each item and for each analytic step is available on the PROMIS website [12].

## Results

### Qualitative activities

#### Item Identification

We identified 329 instruments from a wide range of fields, including cardiology, gastroenterology, rheumatology, neurology, oncology, ophthalmology, pediatrics, and physical medicine and rehabilitation. About half (n = 165) of the instruments contained items that assessed some aspect of physical function. Other instruments measured constructs not relevant to Physical Function, such as pain, fatigue, and satisfaction. From 32 of these instruments, we identified 1,860 potential items, which included Legacy HAQ-DI and PF-10 items. These items composed the initial pool that underwent evaluation.

#### Item Framework

Ninety-three percent (n = 1,728) of the 1,860 items represented one or more of the physical function subdomains. Most of the items (n = 1,578) assessed "capability" ("Are you able..."). The bulk of the remaining items assessed "performance" ("Did you ..."). All 1,860 items were retained in a descriptive file denoting reasons for elimination, intellectual property status, and original item source.

#### Recall Period

We identified 10 different recall periods, which varied widely between and within instruments. More than half of the 1,860 items (n = 975) did not specify a recall period or were prefaced with "the present", "today", or "now". About one fifth (n = 395) used the past week; 15%, (n = 286) the past month; 8% (n = 142), the past 2 weeks; and 3% (n = 48), the past 2 days. The remaining recall periods ranged from 2 months to 1 year.

#### Response Scales and Options

We identified 262 uniquely worded response scales with large variations between and within instruments in the types and numbers of response options and in the placement of extreme responses. Substantial variation was found in how instruments assessed physical function. They ranged from asking about the presence or absence (for example, yes/no), degree of severity (for example, none to extreme), frequency (for example, never to always), to ability (for example, without any difficulty to unable to do).

The number of response options ranged from two (for example, yes/no) to 101 (0 to 100 on a visual analog scale (VAS)). Nearly half (49%) contained four to five response options, which was consistent with study cohort subjects' top two preferences. More than two thirds of the items (n = 1,270) placed the most-negative response (e.g., "unable to do") at the far right end or bottom of the scale), which was also preferred by 71% of the 62 cohort study respondents queried on this item.

#### Binning and Winnowing

We created 71 different bins. The largest proportion of items assessed walking (17%; n = 309) and dressing and grooming (7%; n = 133). Other common bins included eating, gripping, and hygiene. Winnowing eliminated 551 items that were redundant or duplicate, 447 that were condition specific or ungeneralizable, 329 that were confusing or unclear, and 332 that were unrelated or inconsistent with the Physical Function definition. The remaining items were eliminated for miscellaneous reasons. Subjects from the study cohort then evaluated the surviving items.

#### Item Clarity

Subjects assessed whether an item stem or context influenced clarity. Table 1 shows pooled responses for four selected typical physical-function items in which the only difference was that item stems and response options were varied. The Table displays six stems and the response sets of the 12 tested, including the two that were most clear and the two that were least clear.

**Table 1:** Pooled responses about clarity to four physical-function items^a^

Item stem and response options		Number	% Rated unclear (SE^b^)
Are you able to [...]		258	7.75 (0.02)
1. Without any difficulty 2. With a little difficulty 3. With some difficulty	4. With much difficulty 5. Unable to do)		
			
Does your health now limit you in [...]		270	7.78 (0.02)
1. Not at all 2. Very little 3. Somewhat	4. Quite a lot 5. Cannot do		
			
Due to my health [...] is		270	10.74 (0.02)
1. Impossible 2. Very difficult 3. Slightly difficult	4. Easy 5. Very easy		
			
How easy is it for you to [...]		258	11.63 (0.02)
1. Very easy 2. Easy 3. Slightly difficult	4. Difficult 5. Very difficult 6. Impossible		
			
For me, [...] is ...		258	15.12 (0.02)
1. Very easy 2. Easy 3. Slightly difficult	4. Difficult 5. Very difficult 6. Impossible		
			
Does your health now limit you in [...]? If so, how much?^a^		136	17.65 (0.03)
1. Yes, limited a lot 2. Yes, limited a little	3. No, not limited at all		

^**a**^Bathe and dress yourself, climb several flights of stairs, open a new milk carton, and run errands and shop.

^b^Standard error.

^c^PF-10 items, normally presented in grid format, were originally presented as single items with two-part questions. Only item stems and response options varied.

We present results in ascending order of the proportion of subjects rating the item unclear. We found that minor stem or response-option variations often made notable differences. When we compared responses with different stems or response options, subjects better understood simple and straightforward structures. They indicated that items requiring single judgments were clearer than items requiring more than one judgment (for example, "yes, limited a little").

#### Personal Importance and Relevance

Responses regarding personal importance and relevance were highly variable, although results were coherent and logical. Subjects reported that basic activities of daily living (ADLs) like eating, dressing, walking, and self-care were the most important and most relevant. The least important and least relevant items were also the most difficult or complex (for example, "jogging or running 2 miles" or "dancing vigorously for half an hour").

#### Comparison of Legacy and Revised Legacy Items

Legacy HAQ-DI items were originally prefaced with "Over the past week, are you able to ...?" with four response options ("Without any difficulty, with some difficulty, with much difficulty, "unable to do"). Based on findings from the qualitative evaluation, they were revised by deleting the 1-week period, retaining the present tense, and adding one response option ("with a little difficulty"), resulting in a five-point (0 to 4) response scale.

The Legacy PF-10 items were originally preceded by the stem, "The following questions are about activities you might do during a typical day. Does your health now limit you in these activities? If so, how much?" The three response options were "Yes, limited a lot", "Yes, limited a little", and "No, not limited at all". Based on findings, they were rewritten by retaining the first part of the stem ("Does your health now limit you in..."), reversing the scale direction, and increasing the response options to five: "Not at all, very little, somewhat, quite a lot, cannot do".

Table 2 shows selected differences in responses between the Legacy and Rewritten PF-10 and the Legacy and Rewritten HAQ-DI items. We present five items from each instrument, including the easiest and the most difficult. The mean item score represents the instrument mean. Modifying the response options by adding an additional category showed a statistically significant reduction in ceiling effects between the Legacy and rewritten items in both the HAQ-DI (66.3% *versus *73.2%; *P *< 0.01) and the PF-10 (44.9% versus 35.6%; *P *< 0.05).

**Table 2:** Differences in responses to extremes of response options between selected legacy (original) and rewritten PF-10a^a ^and HAQ-DI^b ^items

		PF-10				
	**Legacy**^c^			**Rewritten**^d^		
	Original Stem: During a typical day does your health now limit you in ... If so, how much?			New Stem: How much does your health now limit you ...		
Items	Total Completing Item (n)	Yes, limited a lot (%)	No, not limited at all (%)	Total Completing Item (n)	Cannot do (%)	Not at all (%)
1. Bathing and dressing yourself	81	0	65	74	4	61
2. Climbing one flight of stairs	77	8	55	71	24	41
3. Walking several hundred yards	75	20	55	81	19	48
4. Walking more than a mile	81	32	33	78	31	22
5. Vigorous activities (running, lifting heavy objects, participating in strenuous sports						
Mean instrument score: All 10 PF-10 items		19.8	44.9		23.6	35.6*
		**HAQ-DI**				
	**Legacy^c^**			**Rewritten^d^**		
	**Original stem: Over the past week, have you been able to ...**			**New stem: Are you able to ...**		
	**Total completing item** **(n)**	**Unable to do (%)**	**Without any difficulty (%)**	**Total completing item** **(n)**	**Unable to do** **(%)**	**Without any difficulty (%)**
1. Lift full cup or glass to mouth	81	0	91	78	0	88
2. Climb up five steps	81	2	69	75	4	71
3. Get in and out of a car	71	3	61	71	1	54
4. Reach and get down a 5 lb. object from above your head	78	10	62	81	9	48
5. Chores such as vacuuming/yard work	74	18	35	71	21	30
Mean instrument score: All 20 HAQ-DI items		4.8	73.2		4.7	66.3**

^a^PF-10: 10-item physical function scale of the SF-36.

^b^HAQ-DI: Health Assessment Questionnaire Disability Index.

Response option sets:

^c^Legacy PF-10: Yes, limited a lot; yes, limited a little; no, not limited at all

^d^Rewritten PF-10: Not at all, very little, somewhat, quite a lot, cannot do

^e^Legacy HAQ-DI: Without any difficulty, with some difficulty, with much difficulty, unable to do

^f^Rewritten HAQ-DI: Without any difficulty, with a little difficulty, with some difficulty, with much difficulty, unable to do

* *P *< 0.01; ** *P *< 0.05 between Legacy and Rewritten items.

Table 3 shows subjects' appraisal of clarity between the Legacy and Rewritten HAQ-DI and PF-10 items, and between performance and capability items. The mean proportion of subjects reporting individual items to be unclear ranged from zero to more than 60%, although overall, the proportions reporting them as unclear were relatively low (4.5% to 15.5%). Results showed that rewriting the Legacy items in both the HAQ-DI and PF-10 resulted in substantial improvement (5.4% and 10.8%). Both the rewritten HAQ-DI and PF-10 items were similarly clear, with only about 5% reported as unclear. Subjects also confirmed that present tense "capability" stems were clearer than past tense "performance" stems (8.9% versus 15.5%).

**Table 3:** Appraisal of clarity between legacy (original) and rewritten HAQ-DI^1 ^and PF-10^2 ^and "performance" and "capability" items

Item group (number completing)	**% Unclear (SE**^c^**)**
HAQ-DI, Legacy (20)	5.8 (0.56)
HAQ-DI, Rewritten (20)	5.4 (0.68)
PF-10, Legacy (10)	12.5 (1.6)
PF-10, Rewritten initial (10)	10.8 (1.1)
PF-10, Rewritten final (2)	4.5 (1.8)
Capability ("Are you able...?") (219)	8.9 (0.43)
Performance ("Did you...?") (48)	15.5 (1.2)

^a^Health Assessment Questionnaire Disability Index.

^b^10-item physical function scale of the SF-36.

^c^Standard error of proportion.

#### Item framework: "Capability" versus "Performance" Items

We compared six pairs of typical physical function items, varying only the stem to assess "capability" or "performance". Table 4 shows the pooled analysis comparing responses at the response set's extreme ends (that is, subjects who were unable to perform an activity compared with those who had no difficulty performing an activity). A wide range of subjects was unable to participate in activities, but the mean values between "capability" and "performance" responses were similar (52.8% *versus *50.2%; *P *> 0.05). For most items, there was little difference. Only with the item "Use a hammer to pound a nail", were the responses significantly different (*P *< 0.01). A higher proportion of subjects endorsed that they were able to pound a nail compared with those who said that they had actually performed the activity.

**Table 4:** Worst and best responses to selected capability and performance items by using the identical response set^a^

	Capability: (Present tense -- "Are you able to ...)			**Performance**: (Past tense -- "Did you ...)		
	Number	Unable to do [Worst] (%)	Without any difficulty [Best] (%)	Number	Unable to do [Worst] (%)	Without any difficulty [Best] (%)
Get in and out of bed	75	0	84	75	0	87
Walk outdoors on flat ground	78	8	65	81	4	60
Walk a block	78	9	56	81	10	59
Use a hammer to pound a nail	81	7	72	78	8	50^b^
Climb several flights of stairs	78	18	35	81	19	37
Run or jog 2 miles	81	78	5	78	71	8
						
Mean percentage, all items		20.0	52.8		18.7	50.2
Ratio of worst/best function response			38%			37%

^a^Without any difficulty; With a little difficulty; With some difficulty; With much difficulty; Unable to do

^b^*P *< 0.01 difference between capability and performance item.

### Quantitative analyses

In addition to completing items from the Physical Function item bank, 716 subjects from the PROMIS sample responded to Legacy HAQ-DI and PF-10 items, and 20,376 subjects completed the physical function global health item. For the initial 168 newly developed physical function items, we had responses from more than 2,200 of these subjects. To estimate the item parameters for the Legacy HAQ-DI and the PF-10 items, we supplemented the PROMIS sample with subjects from the study cohorts to allow more stable estimates.

To evaluate construct dimensionality, we started with our *a priori *assumption of four categories. We assigned items to one of the four categories described earlier. A four-factor confirmatory factor analysis showed that within the PROMIS samples, all four factors were highly correlated, which encouraged us to pursue models that are more parsimonious. Even a two-factor solution repartitioning upper and non-upper items correlated highly (*r *> 0.90). Thus, the PROMIS working group made a preliminary decision to include a one-factor solution, recognizing that the final CAT will require some content balancing to avoid underestimation of impairment in those with isolated or regional problems (for example, hand osteoarthritis).

During analysis, we excluded one item in an item pair with residual correlations greater than 0.25 in the confirmatory factor analysis and evaluated all item-response curves heuristically for their monotonicity. For 10 items, we collapsed response categories to achieve a minimal count in any response category of three or to achieve fit to a rank-scaled IRT model, excluding items because of local dependence, insufficient item fit, and DIF. An additional 44 items were excluded, including 22 items that were not intended for calibration (they were device, experimental, or performance items),21 other items, and one item because we were unable to reach the author to obtain permission for use, resulting in 124 of the 168 newly developed items being available for item-parameter estimation.

This core set of items showed good item fit. By using these item-parameter estimates, we estimated the mean and standard deviation (SD) of the physical function score for the "general population," defined by a subset (n = 2,542) of the Polimetrix data who had sociodemographic characteristics similar to the general U.S. population.

We then recalibrated the item parameters to achieve a "general population" mean of 50 and an SD of 10. To estimate item parameters for the Legacy HAQ-DI and PF-10 items, PROMIS items that were similar to the Legacy items were set to missing.

**Figure 2:**
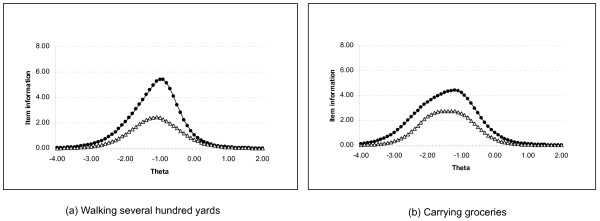
**Information content for two typical physical-function items**. The figures indicate that rewritten items (dashed lines) have improved information content compared with their Legacy counterparts (solid lines). Item information is a function of the area under the curve. A theta of zero represents the population mean (x-axis). Each integer is one standard deviation above or below the mean. More-severe illness is represented by points to the left of zero.

Figure 2 displays the item information content for two typical Legacy (in solid lines) items (running errands and carrying groceries) and their revised counterparts (in dashed lines). The Theta of zero represents the study population mean. Each integer is one standard deviation above or below that population mean. Data to the left of zero indicate poorer physical function. The data show that the revised items have much higher item-information content than do the corresponding Legacy items.

## Discussion

This initial study applied systematic modern item-assessment methods to develop the PROMIS Physical Function item bank. These results demonstrate that improved physical function items could be developed by using comprehensive qualitative and quantitative item-evaluation procedures. The PROMIS Physical Function item bank is an important advancement in the assessment of physical function and is available for use in studies through the PROMIS Assessment Center^sm ^[12].

New, important, and striking findings emerged from these item analyses. First, items that were framed as "ability" (that is, what a person "can do", which taps into limitations or strengths of doing something) rather than "performance" (that is, what a person "does do", which refers to a frequency of task performance) to assess physical function strongly predominated. Although tabulation of popularity is an imperfect approach, absent evidence to the contrary, it lends substance to continue existing practice. Second, basic activities of daily living (for example, walking, dressing) were more relevant and important to laypersons with arthritis and people who average 70 to 80 years of age compared with more-difficult and complex activities (for example, dancing or jogging). Thus, at an absolute minimum, Physical Function scales must include basic activities. Third, the present tense, a simple sentence structure, and straightforward uncomplicated questions enhance clarity and comprehension. Fourth, responses that contain four or five options rather than two or three provide greater discrimination and reduce floor/ceiling effects over a broad range of measurement.

The Physical Function domain satisfies general criteria for unidimensionality, with one-, two-, three-, and four-factor models having comparable model fits, and with correlations between factors being very high in the test data sets (*P *> 0.90). However, a conceptual and practical problem suggests that this may not be true in all data sets or in an individual. The majority of subjects in these analyses had relatively little disease burden compared with that of typical clinical trial subjects. The mobility (lower extremity) and activities factors tend to dominate over the other potential factors (upper, axial, activities) in short forms developed from item parameters and CAT applications. However, a unidimensional model might not be a valid representation when disability is concentrated in the hand (such as in scleroderma or hand osteoarthritis) or in the axial regions (such as in ankylosing spondylitis or cervical spine disease). Additional PROMIS projects are currently under way, addressing this issue. Because nothing is lost (except simplicity), and face validity may be enhanced and individual subjects better described, a strong argument can be made for using the four-factor model until sufficient populations have been studied to assess the magnitude of these issues. At the least, to assure input from each potential subdomain, we need content balancing to approach face validity problems.

The strengths of this study include its sheer size and effort and its protocol-driven, comprehensive item-level evaluation. Item-improvement processes have seldom been undertaken so broadly and in such detail, with careful attention to clarity and personal relevance. Limitations include the relatively small number of rheumatic disease subjects in this pool of available subjects, a lower-than-desirable sample of clinically severe subjects, and the lack of ethnic diversity. In addition, we used only mailed questionnaires for qualitative assessments. We might have obtained different results if we had a different subject base or used other modes of administration, such as the Internet, telephone, or a hand-held device.

This study shows that item improvement must underlie attempts to improve outcome assessment. The use of IRT and CAT has advanced PRO assessment so that floor/ceiling effects can be reduced, measurement precision, efficiency, validity, and meaningful results can be increased, and subject burden reduced. However, this has not yet become a standard approach in medicine, although item banking and IRT are conventional methods in fields such as educational testing [6].

## Conclusions

The clear, personally important and relevant, ability-framed items in the PROMIS Physical Function item bank perform well in PRO assessment. They will benefit from further study and application in a wider variety of rheumatic diseases, in diverse clinical groups including those at the extremes of physical functioning, and in different administration modes.

## Abbreviations

ADL: basic activities of daily living; ARAMIS: Arthritis, Rheumatism, and Aging Medical Information System; CAT: computerized adaptive testing; HAQ-DI: Health Assessment Questionnaire Disability Index; IRT: item response theory; PF-10: SF-36 Health Survey's Physical Function scale; PRO: patient-reported outcome; PROMIS: Patient Reported Outcomes Measurement Information System; PROQOLID: Patient Reported Outcomes Quality of Life Instruments Database; VAS: visual analog scale.

## Competing interests

The authors declare that they have no competing interests.

## Authors' contributions

BB, JF, MR, BG and JW contributed to conception and design, and participated in data analysis and interpretation, and drafting and reviewing of manuscript. DA participated in data collection and analyses. BL participated in data analysis.
